# Design and Testing of an Integrated Robot for Harvesting, Stipe-Cutting, and Grading of *Agaricus bisporus*

**DOI:** 10.3390/s26144414

**Published:** 2026-07-11

**Authors:** Tianhang Ding, Yingying Zhou, Hao Ma, Yulong Ding, Hongwei Cui

**Affiliations:** 1 Key Laboratory of Modern Agricultural Equipment, Ministry of Agriculture and Rural Affairs, Nanjing 210008, China; hangtian1888@163.com; 2 College of Agricultural Equipment Engineering, Henan University of Science and Technology, Luoyang 471000, China; 250320261683@stu.haust.edu.cn (Y.Z.); mahao@haust.edu.cn (H.M.); 3 Zhejiang Key Laboratory of Intelligent Sensing and Robotics for Agriculture, College of Biosystems Engineering and Food Science, Zhejiang University, 866 Yuhangtang Road, Hangzhou 310058, China; 4 Chinese Academy of Agricultural Mechanization Sciences Group Co., Ltd., Beijing 100083, China; ding18437910053@163.com

**Keywords:** *Agaricus bisporus*, deep learning, harvesting robot, stipe-cutting, grading

## Abstract

To address the limitations of current *Agaricus bisporus* harvesting robots, including low picking efficiency, susceptibility to mechanical damage, poor operational stability, and discontinuous harvesting processes, an integrated robotic system capable of mushroom detection and localization, picking sequence planning, stipe-cutting, and grading was developed. The robot adopts a left-right symmetrical configuration and operates along the side of the mushroom cultivation racks. It mainly consists of a mobile platform, a lifting mechanism, dual picking units, a receiving unit, a stipe-cutting device, a collection system, and an electronic control system. A picking sequence planning method based on YOLOv8n-USD and KD-tree nearest neighbor search was employed to determine the optimal harvesting order for densely clustered and adhered mushrooms. The dual robotic arms executed the picking operations, after which the mushrooms were transferred to the receiving unit for stipe-cutting and subsequently classified according to the detection results, thereby completing the integrated process of detection, picking, stipe-cutting, and grading. Experimental results show that the average detection accuracy reaches 96.64%, the picking success rate is 95.39%, and the harvesting damage rate is 1.63%, while the average interaction failure rate and grading error rate are 0.35% and 1.28%, respectively. These results indicate that the proposed robot can achieve accurate detection and efficient integrated operation for densely clustered *Agaricus bisporus*. The findings provide a reference for flexible and efficient harvesting of *Agaricus bisporus* with synchronized stipe-cutting and grading operations.

## 1. Introduction

With the development of large-scale and intelligent agricultural production, factory-based cultivation of edible fungi has become the mainstream production mode [1]. The edible fungi industry is an important component of China’s agriculture and also serves as a key sector for poverty alleviation in underdeveloped regions [2]. In the future, it is necessary to accelerate factory-based production, enhance international market competitiveness, and promote the further development of the edible fungi industry in China [3,4]. The *Agaricus bisporus*, known as the “world mushroom”, is the most widely cultivated, largest-scale-produced and highest-yielding edible fungus in the world, accounting for approximately 95% of the consumption market [5]. It has a nutritional value 4–12 times higher than that of fruits and vegetables and is known as the “king of vegetarian foods” [6]. It also exhibits certain medicinal functions and is highly favored in both domestic and international markets [7].

In recent years, with the widespread adoption of factory-based cultivation technologies, the production of *Agaricus bisporus* has shifted from decentralized cultivation to centralized management, with multi-layer cultivation beds and enclosed mushroom houses becoming the dominant configuration [8]. The mushroom cap is soft, and uneven force during harvesting can easily cause cap damage and stipe residue, which not only directly affects market value but also influences subsequent production cycles [9]. In practical production, the harvesting window is short, and a large number of mature mushrooms must be processed within a few hours, placing high demands on efficiency and operational stability [10]. At present, most mushroom houses still rely on manual harvesting, and maintaining timely picking and freshness requires substantial labor and material input [11]. Manual harvesting and grading are characterized by low efficiency, high labor intensity, high labor costs, and significant damage, which severely affect production efficiency and product quality [12].

To overcome these limitations, extensive research on *Agaricus bisporus* harvesting robots has been conducted worldwide. The most advanced mushroom harvesting robot developed in Germany employs multi-unit coordination to automatically scan, harvest, cut the stipe, and place mature mushrooms, enabling continuous 24-h operation [13]. A side-mounted structure proposed in Canada provides a certain degree of flexibility. However, its design primarily focuses on the harvesting function and does not integrate harvesting, stipe-cutting, and grading operations within a single robotic platform [14]. In China, the technological and industrial foundations of agricultural robotics are continuously improving [15], and several approaches, including gantry-type, SCARA-type, and biomimetic flexible grippers, have gradually been developed [16,17], with some systems achieving integration of stipe-cutting and grading functions. A robot developed by Shanghai University divides the system into vision, harvesting, and auxiliary zones and adopts a “three-step” strategy, in which inspection is followed by harvesting and operations are completed through zonal coordination [18]. Nanjing Agricultural University [19] developed a mobile anthropomorphic harvesting robot equipped with dual SCARA arms, capable of side lifting and integrated operations of detection, harvesting, and stipe-cutting. However, due to the dense and adherent growth characteristics of *Agaricus bisporus*, challenges remain in determining appropriate picking sequences based on growth height and density to reduce harvesting damage. In addition, after stipe-cutting, achieving automated grading to reduce secondary damage caused by manual sorting still requires further investigation [20].

Accurate identification of *Agaricus bisporus* using computer vision is a prerequisite for mechanized harvesting. Traditional digital image processing methods can achieve segmentation and recognition of mushrooms in complex backgrounds [21,22]. However, for densely clustered and adherent mushrooms, these methods suffer from limited real-time performance and cannot meet the requirements of robotic harvesting. Deep learning-based detection algorithms utilize neural networks to accurately identify targets [23,24,25], improving detection performance, but they do not address the planning of picking sequences. Without a reasonable harvesting sequence, even with high detection accuracy, adjacent mushrooms may be damaged during the picking process due to improper operation order [26]. Compared with traditional image processing methods, YOLOv8n-USD is a lightweight one-stage detection and segmentation framework that combines real-time inference capability with enhanced feature extraction for dense and overlapping mushroom targets. By improving multi-scale feature representation and boundary perception, it achieves accurate target localization while satisfying the computational constraints of edge devices used in harvesting robots. A KD-tree (k-dimensional tree) is a spatial partitioning data structure designed for efficient nearest-neighbor search in multidimensional space. In robotic harvesting applications, it enables fast organization and retrieval of three-dimensional target coordinates, allowing mushrooms to be associated according to their spatial proximity [27].

Existing mushroom harvesting robots are often limited by single-function operation, insufficient integration of harvesting-related processes, and the lack of harvesting sequence planning in dense cultivation environments. To address these challenges, an integrated robotic system for *Agaricus bisporus* harvesting, stipe-cutting, and grading was developed. The proposed system combines visual recognition and localization, harvesting sequence planning, robotic harvesting, stipe-cutting, and grading functions within a unified platform, enabling the automated execution of the complete harvesting workflow. 

The structure of this paper is arranged as follows. The hardware design of the proposed robotic harvesting system is presented in Section 2. The identification method and harvesting sequence planning algorithm for *Agaricus bisporus* harvesting are described in Section 3. The control system design, including motion control and system integration, is presented in Section 4. The system experiments and performance evaluation results are reported in Section 5. A comparative discussion with existing harvesting robots is provided in Section 6, where the advantages, limitations, and future research directions of the proposed system are further discussed. Finally, the conclusions of this study are presented in Section 7.

## 2. The Hardware Design

### 2.1. Harvesting Requirements Analysis of Agaricus bisporus

**Figure 1:**
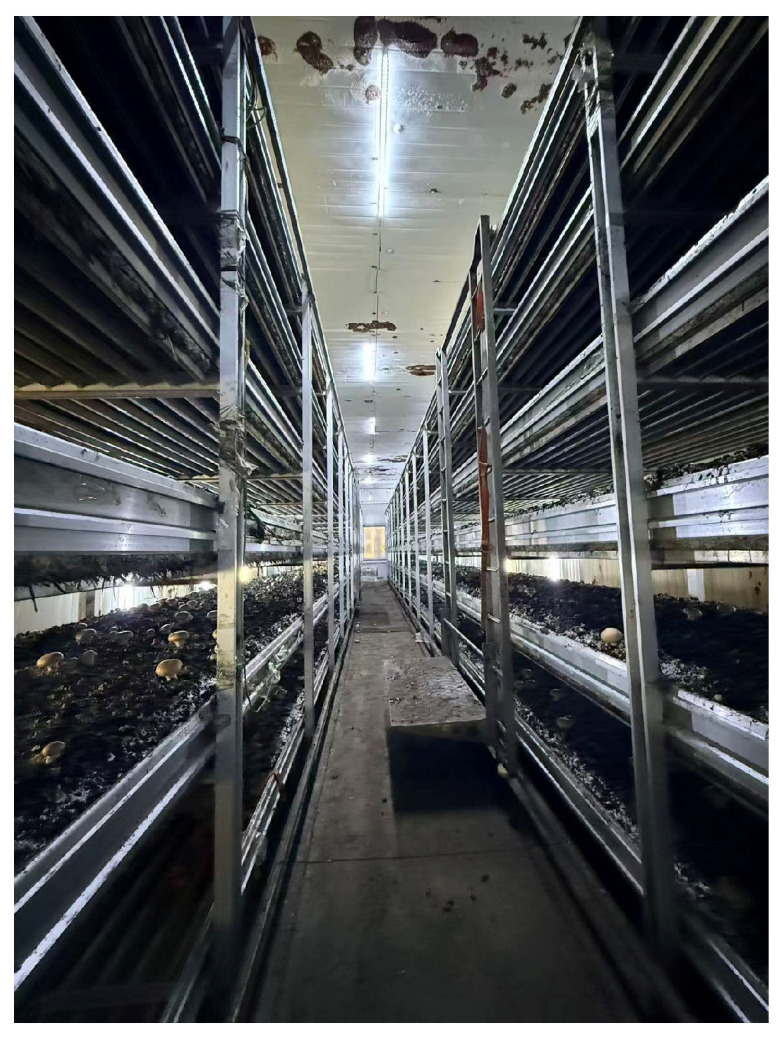
The standard mushroom house.

Modern *Agaricus bisporus* cultivation factories typically adopt sterile mushroom rooms, where environmental factors such as temperature, humidity, and illumination are regulated through intelligent control systems to provide suitable growing conditions. In this study, a standard mushroom house of Luoyang Aojite Agricultural Development Co., Ltd. was taken as the design reference. The mushroom house is equipped with two rows of cultivation racks, each constructed with a multi-layer gantry structure. The spacing between adjacent racks is 1480 mm, and each rack contains six layers of cultivation beds with an interlayer height of 400 mm. This configuration makes efficient use of space while meeting the growth requirements of *Agaricus bisporus* and also provides structural support for automated production, ensuring both production efficiency and sustainability, as shown in Figure 1.

Considering the harvesting process requirements and environmental constraints, the following design requirements are proposed for the harvesting robot.

- The harvesting system must operate within the limited space of the mushroom house and cultivation layers to accomplish movement, lifting, picking, grasping, stipe-cutting, and grading. This requires a compact overall structure to adapt to the confined workspace, while also imposing constraints on the working range of the robotic arm and the installation position and height of the depth camera.
- *Agaricus bisporus* exhibits dense and adherent growth, with individual fruiting bodies often occluding each other. Therefore, the identification algorithm must achieve accurate detection and localization and should also determine appropriate picking sequences for clusters with different density levels to reduce damage to adjacent mushrooms.
- The cap diameter of mature *Agaricus bisporus* is typically 30–60 mm. The mushroom surface is soft and easily damaged, and the fruiting body is connected to the substrate through the stipe. Direct vertical pulling may cause tearing or residue. Therefore, the end-effector of the harvesting robot should enable flexible picking and incorporate a rotational motion to gradually detach the stipe from the substrate, thereby reducing the damage rate.

### 2.2. Overall Structural Design

**Figure 2:**
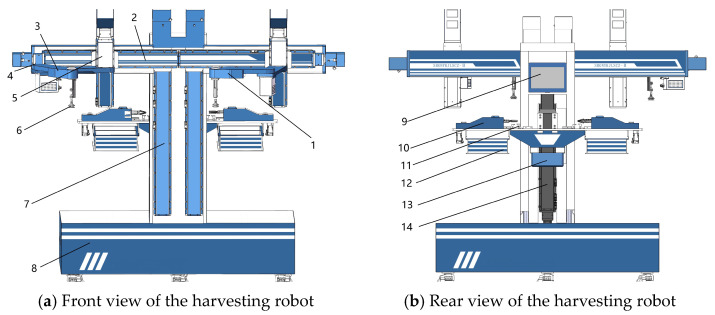
*Agaricus bisporus* harvesting device diagram. 1. Depth camera. 2. Horizontal sliding module. 3. Forearm link. 4. Upper arm link. 5. Lifting module of the robotic arm. 6. Flexible suction cup. 7. Picking lifting module. 8. Mobile platform. 9. Human–machine interface. 10. Receiving unit. 11. Stipe-cutting device. 12. Mushroom collection unit. 13. Stipe residue collection unit. 14. Lifting module of the receiving unit.

To adapt to the constrained operating space in the mushroom factory room, the overall mechanical structure of the *Agaricus bisporus* harvesting robot is shown in Figure 2 (detailed descriptions are provided below). 

The system adopts a symmetrical configuration and mainly consists of a mobile platform, a lifting mechanism, a picking unit, a receiving unit, a stipe-cutting device, and a collection unit. The mobile platform serves as the base carrier of the robot and is composed of wheels, a housing, and electrical components. It enables movement between cultivation racks and accurate positioning at the designated reference location. The lifting mechanism includes a picking lifting module and a collection lifting module, which are mainly composed of embedded linear slide modules and servo motors. The picking lifting module allows the robotic arm to reach different cultivation bed heights, while the collection lifting module carries the receiving unit, stipe-cutting device, and collection unit to move synchronously with the picking module.

The picking unit consists of a planar two-link robotic arm, a flexible suction cup, a vertical lifting module, and a horizontal sliding module. The planar two-link arm equipped with a flexible suction cup can move within the narrow cultivation space. After the suction cup contacts the *Agaricus bisporus*, the mushroom is separated from the substrate through pressure variation, and the vertical motion of the robotic arm is achieved by the lifting module to complete the picking operation. The receiving unit is composed of a receiving gripper, a receiving arm, and a motor. The harvested mushroom is first placed onto the receiving gripper, which then transfers it to the stipe-cutting device for subsequent cutting and grading operations. The stipe-cutting device consists of two parallel cutting blades, which are used to perform uniform stipe-cutting. The collection unit includes two mushroom collection bins for different grades and one waste bin for stipe residues, which are used to store harvested mushrooms and removed stipes.

The mechanical structure was designed to achieve efficient harvesting, stipe-cutting, grading, and collection of Agaricus bisporus, thereby meeting the requirements of automated production.

### 2.3. Key Structural Design

#### 2.3.1. Design of the Lifting Mechanism

Gantry-type harvesting robots are susceptible to collisions with cultivation structures and adjacent mushrooms, while their limited workspace restricts harvesting at bed edges. To overcome these limitations, a side-mounted harvesting configuration with a lifting mechanism was adopted, enabling flexible harvesting across different cultivation layers.

The lifting mechanism consists of a picking lifting module and a receiving lifting module. The picking lifting module adjusts the height of the picking unit through an embedded linear slide module and a servo motor, allowing flexible positioning across different cultivation layers. The receiving lifting module follows the vertical position of the picking module and adjusts the height of the receiving unit, stipe-cutting device, and collection unit accordingly, ensuring the completion of harvesting, stipe-cutting, and grading operations. In this study, embedded linear slide modules are employed. These modules are driven by ball screw mechanisms, which feature low friction and high transmission efficiency, enabling high-precision linear motion. The repeat positioning accuracy can reach ±0.005 mm, ensuring stable and reliable long-term operation of the lifting mechanism.

#### 2.3.2. Design of the Picking Unit

**Figure 3:**
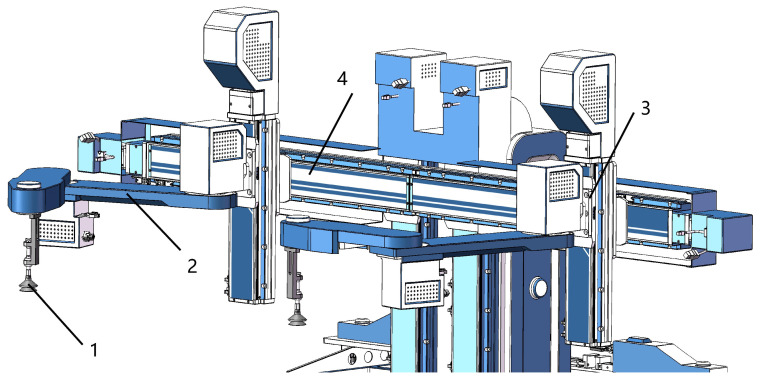
Harvesting device structure diagram. 1. Flexible suction cup. 2. Robotic arm. 3. Lifting module of the robotic arm. 4. Horizontal module of the robotic arm.

**Figure 4:**
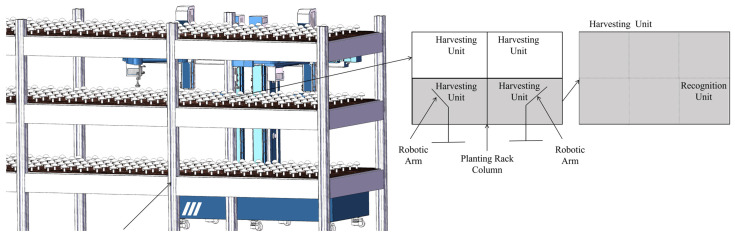
The work zone and division.

The picking unit consists of a flexible suction cup, a robotic arm, a robotic arm lifting module, and a horizontal sliding module, as shown in Figure 3. The harvesting manipulator was designed to harvest half of the cultivation bed width. The upper arm and forearm lengths were 470 mm and 350 mm, respectively, providing a maximum reach of approximately 820 mm. The work zone and division are shown in Figure 4.

Both the horizontal sliding module and the lifting module of the robotic arm are embedded linear slide modules. The lifting module is connected to the horizontal sliding module through a linkage component, enabling the robotic arm to move flexibly along the Y-axis. This configuration reduces the overall length of the picking unit while increasing the effective motion range in the Y-direction. The robotic arm is mounted on the lifting module via a vertical load-bearing support, allowing movement along the Z-axis.

The flexible suction cup is installed at the end of the robotic arm and can cover the entire picking area. To avoid interference with other components during non-operational periods, the robotic arm can retract beneath the horizontal sliding module. During harvesting, the robotic arm extends into the working area and performs picking operations, ensuring efficient and accurate task execution.

- Picking robotic arm

**Figure 5:**
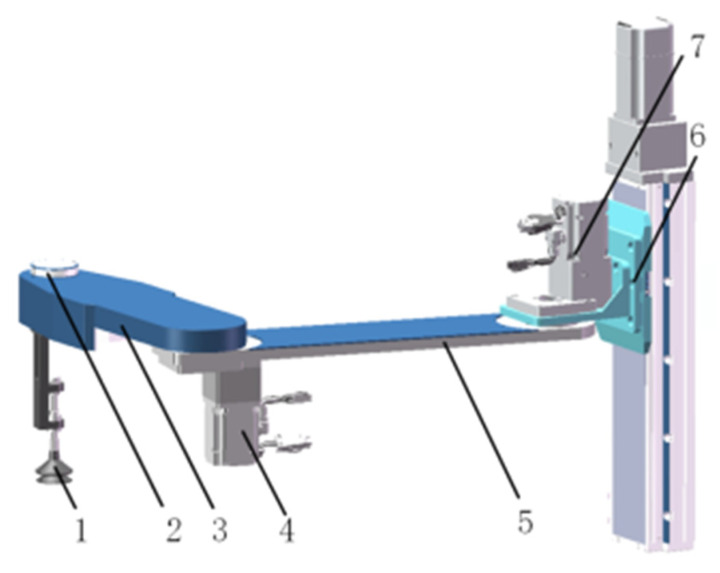
Harvesting robotic arm structure diagram. 1. Flexible suction cup. 2. Swing cylinder. 3. Forearm link of the robotic arm. 4. Forearm motor. 5. Upper arm link of the robotic arm. 6. Connecting component. 7. Upper arm motor.

To adapt to the narrow spacing between cultivation beds in the *Agaricus bisporus* growing environment and to improve the stability and safety of the harvesting process, a planar two-link robotic arm is adopted as the picking manipulator. The arm is connected to the lifting module through a waist support component; the upper arm and forearm are driven by servo motors, and the flexible suction cup is connected to the end of the forearm via a swing cylinder, as shown in Figure 5. The specific parameters are shown in Table 1.

- End-effector

**Figure 6:**
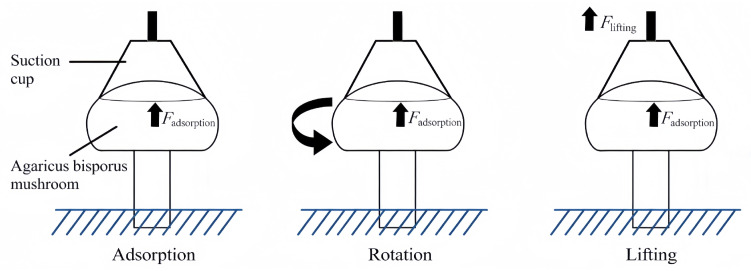
Flexible suction cup harvesting action.

Due to the dense and clustered growth characteristics of *Agaricus bisporus*, a flexible suction cup is selected as the end-effector, and harvesting is achieved through suction-based attachment. The flexible conformal suction cup achieves a harvesting success rate of 98.5% and a damage rate of 2.5% [28]. As shown in Figure 6, the harvesting action is simplified in this study. After contacting the mushroom cap, the suction cup generates pressure and suction force to stabilize the mushroom. By rotating the suction cup, the mushroom rotates around the central axis of the stipe, allowing separation from the substrate. At the same time, the robotic arm lifts the end-effector together with the mushroom to complete the harvesting process.

**Figure 7:**
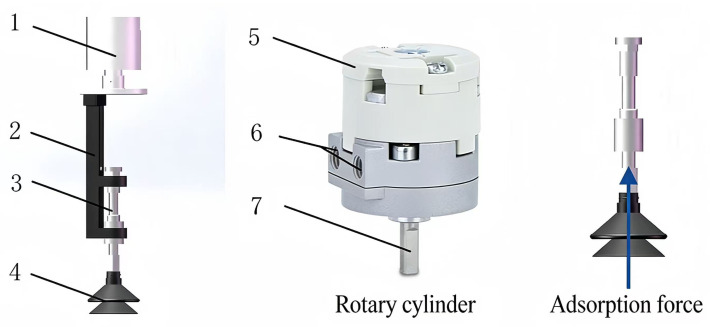
3D diagram of the end effector. 1. Rotary cylinder. 2. Connecting component. 3. Telescopic rod. 4. Flexible suction cup. 5. Magnetic switch slot. 6. Air inlet and outlet ports. 7. Rotating shaft.

The end-effector, shown in Figure 7, consists of a rotary cylinder, connecting components, a telescopic rod, and a flexible suction cup. The negative pressure required for suction is generated by a vacuum pump mounted on the robot base and delivered to the suction cup through a flexible pneumatic tube, thereby reducing the mass and inertia of the end-effector.

To provide quantitative design parameters for the flexible suction cup, the suction force, torque, and contact pressure were analyzed based on our previous study [27] and additional calculations. Under the actual harvesting negative pressure of –9.2 kPa obtained from field tests, the average adsorption force measured using a 35 mm diameter mushroom model was 2.85 N. The theoretical suction force contributed solely by negative pressure is F1=πD2p4=0.88N, and the remaining 1.97 N is provided by friction due to the conformal contact between the flexible membrane and the mushroom cap. The rotary cylinder (CDRA1BS30-90, SMC) installed at the end of the forearm provides a rated torque of 0.45 N·m at an operating pressure of 0.6 MPa, while the experimentally measured torque required for stipe detachment is less than 0.3 N·m, ensuring sufficient torque margin for the twisting separation process. In addition, finite element simulation results [28] indicate that the maximum contact pressure on the mushroom cap is approximately 0.63 kPa under the actual harvesting pressure, which is lower than the damage threshold of approximately 2 kPa and contributes to the low damage rate achieved by the robot.

#### 2.3.3. Structure of the Receiving and Stipe-Cutting Unit

**Figure 8:**
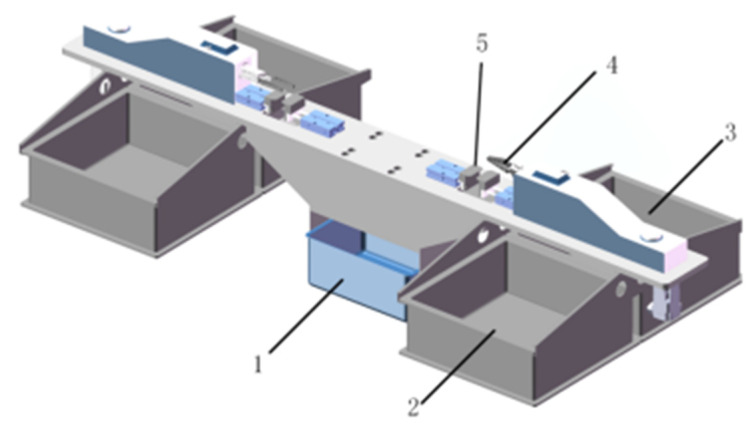
Receiving and collection device. 1. Root collection box. 2. Class A collection box. 3. Class B collection box. 4. Receiving gripper. 5. Trimming blade.

To reduce mutual interference between picking arms during operation and improve overall efficiency, the robot is equipped with an independent receiving arm and a stipe-cutting mechanism, as shown in Figure 8. 

This unit is used to receive *Agaricus bisporus* harvested by the robotic arm and complete subsequent stipe-cutting and grading operations. After the mushroom enters the receiving area, the stipe-cutting mechanism removes the stipe and transfers the mushroom into the corresponding collection bin according to the classification requirements. Meanwhile, the picking robotic arm can move to the next target position to continue harvesting, enabling continuous operation. According to the grading standard of *Agaricus bisporus*, harvested mushrooms are typically divided into two categories. Therefore, two collection bins are configured for each robotic arm to store mushrooms of different grades. In addition, a stipe residue collection bin is arranged to collect the removed stipes for subsequent processing.

- Receiving unit

**Figure 9:**
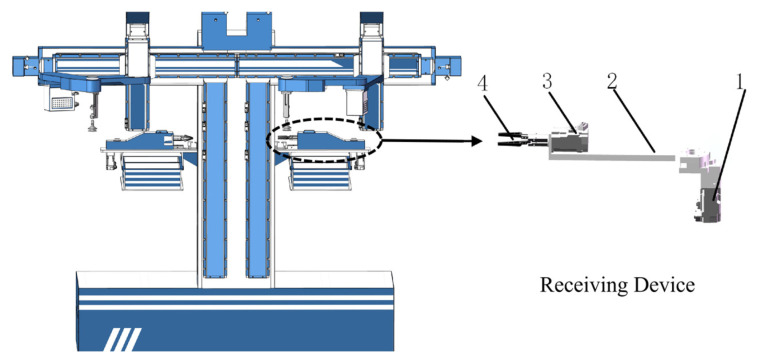
Structure diagram of the receiving device. 1. Receiving arm servomotor. 2. Receiving arm. 3. Servomotor. 4. Receiving gripper.

The receiving unit mainly consists of a receiving gripper, a motor, and a receiving arm, as shown in Figure 9. 

The gripper is responsible for receiving the mushroom picked by the end-effector and transferring it to the stipe-cutting position. After cutting, the servo motor drives the gripper to rotate, allowing the mushroom to be released into the corresponding collection bin for classified storage. After completing the grading process, the receiving unit returns to its initial position to prepare for the next operation.

- Stipe-cutting device

**Figure 10:**
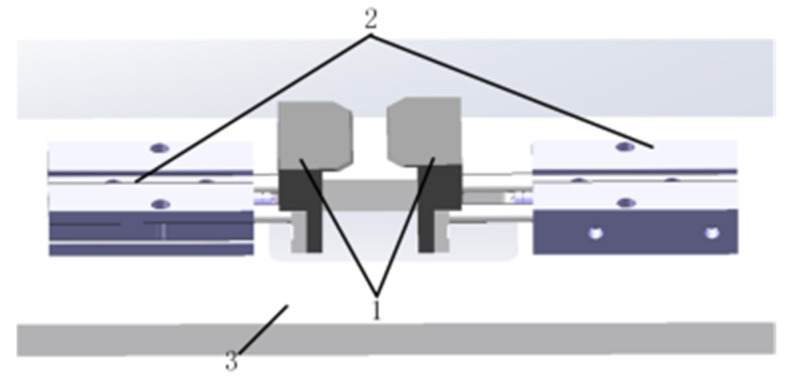
Root cutting device. 1. Dual-rod cylinder. 2. Trimming blade. 3. Receiving platform.

The stipe-cutting device consists of dual cutting blades, a double-rod pneumatic cylinder, and a receiving platform, as shown in Figure 10.

The receiving gripper places the mushroom in the cutting area, and the double-rod pneumatic cylinder drives the parallel blades to perform stipe-cutting by regulating air pressure. The stable force provided by the cylinder ensures consistent cutting performance and a smooth cutting surface.

Since the relative position between the cutting blades and the receiving platform remains fixed, the residual stipe length is controlled by adjusting the transfer height between the harvesting manipulator and the receiving platform. By changing the mushroom release position before cutting, different trimming lengths can be achieved to satisfy processing requirements.

### 2.4. Working Principle of the Harvesting Robot

**Figure 11:**
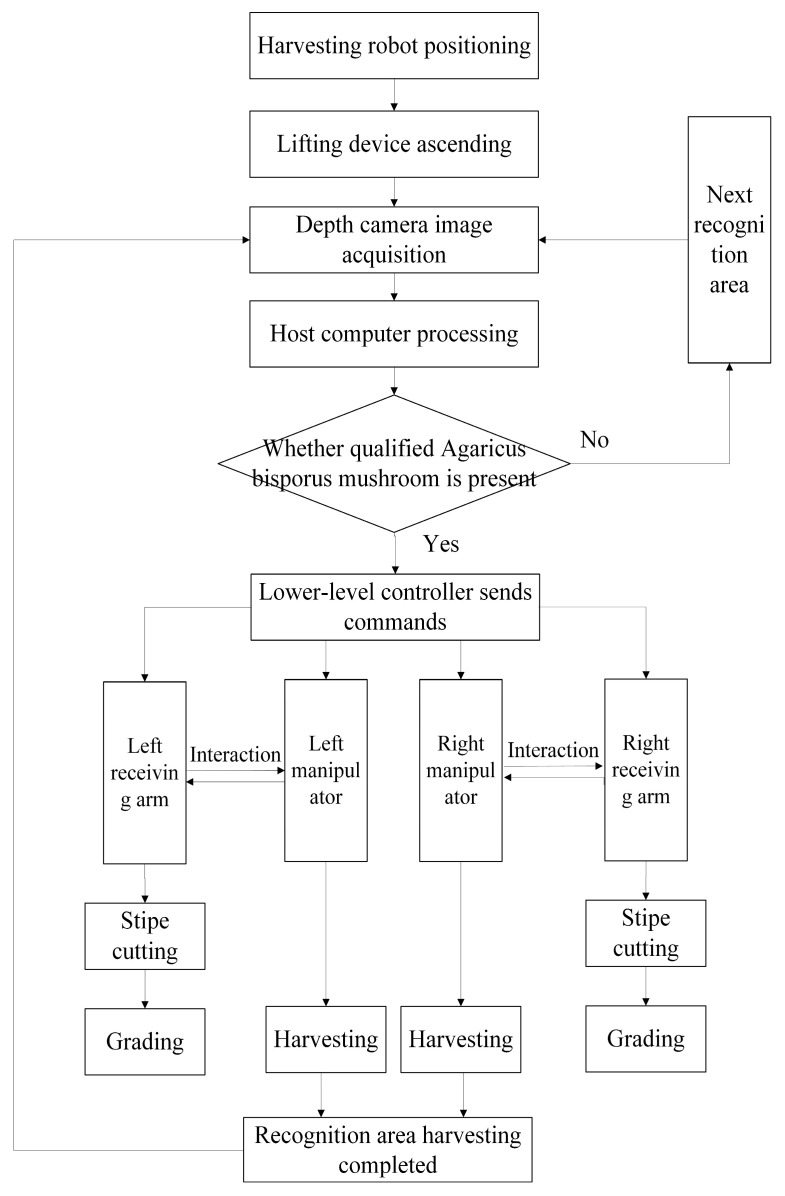
Working principle flowchart.

The operation of the harvesting robot can be divided into five stages, as shown in Figure 11.

In the first stage, the robot moves to one side of the cultivation rack, and the lifting mechanism adjusts the height of the robotic arms to the designated position, with each arm responsible for one picking unit.

In the second stage, mushroom identification, localization, and picking are performed. The robotic arm carries the camera to the target area to capture images, which are transmitted to the industrial computer. The computer processes the images to determine the positions and radii of the target mushrooms and completes the picking sequence planning. The generated data are then transmitted to the motion controller, which interprets the data and issues commands to control the robotic arm and end-effector to execute the picking operation.

In the third stage, stipe-cutting is carried out after harvesting. The end-effector places the harvested mushroom onto the receiving gripper, which transfers it to the cutting position for stipe removal. Meanwhile, the robotic arm proceeds to harvest the next mushroom.

In the fourth stage, mushroom grading is performed. The industrial computer classifies mushrooms into grade A and grade B based on their radii. For grade A mushrooms, the receiving unit rotates clockwise to the corresponding collection bin and releases the mushroom. For grade B mushrooms, the receiving unit rotates counterclockwise and releases the mushroom in the same manner.

In the fifth stage, a missed detection check is conducted. After completing harvesting in the current area, the camera captures images again to determine whether any mushrooms remain unharvested. If so, the robot continues the harvesting process; otherwise, it moves to the next detection area and repeats the above stages.

## 3. Identification and Picking Sequence Planning Algorithm

### 3.1. Growth Characteristics of Agaricus bisporus and Dataset Construction

Under factory cultivation conditions, *Agaricus bisporus* usually grow in batches. Across different flushes, the spacing, maturity, and spatial distribution of mushrooms vary noticeably. During the first flush, mushrooms are densely distributed with obvious overlap. In the second flush, both density and height variation decrease, and overlap is reduced. By the third flush, the height becomes more uniform, and the density further decreases. In the fourth flush, mushrooms are sparsely distributed with relatively uniform height, and individual mushrooms grow independently with almost no overlap.

The image data were collected from a mushroom cultivation base of Luoyang Songtian Agricultural Development Co., Ltd. An Orbbec Gemini2 depth camera was used to acquire both RGB images and depth images of *Agaricus bisporus* across four growth stages to construct the dataset. During data acquisition, the distance between the camera and the mushroom bed was 350 mm. The image resolution was 1280 × 720 pixels, and a total of 875 images were obtained.

The dataset consisted of mushroom images collected under different growth stages, densities, and illumination conditions in the cultivation environment. To ensure dataset diversity and model generalization, images containing dense clusters, sparse clusters, and individual mushrooms were included. The dataset was randomly divided into training, validation, and test sets according to a ratio of 7:1:2, while maintaining a similar distribution of mushroom categories in each subset. To improve model robustness and generalization ability, data augmentation was applied. The training and validation datasets were expanded through brightness adjustment, Gaussian noise addition, motion blur, and image rotation. Finally, the dataset included 2452 training images, 352 validation images, and 174 test images.

According to the growth characteristics, *Agaricus bisporus* were categorized into three types: dense clusters, sparse clusters, and individual mushrooms. Individual mushrooms have no neighboring mushrooms nearby. Sparse clusters consist of 2 to 7 mushrooms with no overlap and small height differences. Dense clusters are characterized by high density and obvious overlap.

### 3.2. Picking Sequence Planning Algorithm

This study adopts a previously proposed method that integrates YOLOv8n-USD with KD-tree nearest neighbor search for mushroom classification and picking sequence planning [26]. In the present study, this method is integrated into the proposed harvesting robot for real-time mushroom classification, segmentation, and picking sequence generation.

**Figure 12:**
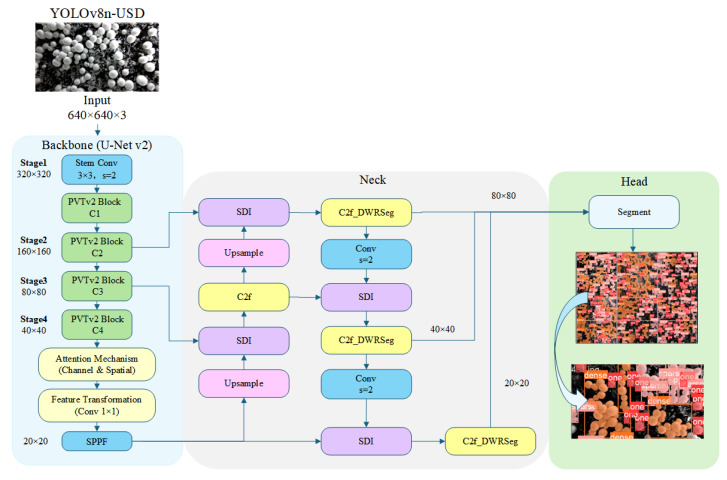
YOLOv8n-USD network structure diagram.

As shown in Figure 12, the proposed YOLOv8n-USD network consists of a U-Net v2 backbone, an SDI-based feature fusion neck, and a segmentation head. The backbone adopts a PVTv2 encoder with four hierarchical feature extraction stages to capture multi-scale representations of mushroom targets. A channel-spatial attention mechanism and feature transformation layer are further introduced to enhance discriminative feature learning and suppress background interference. In the neck, SDI modules are employed to fuse semantic information from deep layers and detailed spatial information from shallow layers. Meanwhile, the original C2f modules are replaced with C2f_DWRSeg modules, which integrate DWR-based contextual feature extraction and SIR-based lightweight feature refinement to improve boundary perception for densely clustered mushrooms. The fused multi-scale features are finally delivered to the segmentation head for target classification and pixel-level segmentation.

The overall workflow of the proposed perception and planning pipeline is summarized as follows. First, the improved YOLOv8n-USD model is used to perform mushroom detection and segmentation, generating instance-level masks with class labels. These segmentation results are then aligned with the corresponding depth images to construct 3D point clouds, providing spatial information for subsequent processing. Next, morphological operations combined with circular feature extraction are applied to refine object contours and obtain the center coordinates of individual mushrooms, which serve as the basis for spatial reasoning in the picking task.

Based on the processed spatial information, a hierarchical picking sequence strategy is applied. Let $P=\left\{p_{1},p_{2},\ldots ,p_{n}\right\}$ represent the set of 3D spatial centroids of the localized *Agaricus bisporus* targets, where each target $p_{i}$ is parameterized by its Cartesian coordinates $\left(x_{i},y_{i},z_{i}\right)$, its growth density category $C_{i}\in \left\{\mathrm{one},\mathrm{sparse},\mathrm{dense}\right\}$, and its depth projection $L_{i}$ relative to the camera baseline. To accelerate local coordinate querying, a balanced 3D KD-tree $\left(T\right)$ is constructed using the coordinate set P, enabling efficient nearest-neighbor queries during spatial target retrieval.

The harvesting sequence is generated through a hierarchical scheduling framework consisting of global and local planning stages. 

At the global level, mushroom targets are first grouped according to their density category and prioritized as

$$\mathrm{Priority}\left(C_{i}\right)=\left\{\begin{array}{c} 1,\;\;\;\;\;\;\;\mathrm{if}\;C_{i}=\mathrm{one}\\ 2,\;\mathrm{if}\;C_{i}=\mathrm{sparse}\\ 3,\;\;\mathrm{if}\;C_{i}=\mathrm{dense} \end{array}\right.$$

where a lower priority value corresponds to a higher harvesting priority.

On the local level, within each category, different scheduling rules are applied according to the spatial morphology of the mushrooms.

1. One category

For isolated mushrooms, the harvesting order is determined according to the Euclidean distance between each mushroom centroid and the camera origin $O_{c}$:

$$D_{i}=\|p_{i}-O_{c}{\|}_{2}$$

Targets are sorted in ascending order of $D_{i}$, and mushrooms closer to the camera are harvested first to reduce manipulator travel distance.

2. Sparse category

For sparse clusters, a rotational harvesting sequence planning strategy is employed. Let $P_{c}$ denote the centroid of the cluster. The radial distance of each mushroom from the cluster center is calculated as

$$R_{i}=\|p_{i}-P_{c}{\|}_{2}$$

Mushrooms are sorted in descending order of $R_{i}$, such that the outermost mushrooms are harvested first, followed progressively by mushrooms located closer to the cluster center. By removing peripheral mushrooms before harvesting inner targets, the algorithm effectively enlarges the available operating space and reduces interference among neighboring mushrooms.

3. Dense category

For dense clusters, harvesting priority is determined according to both height variation and spatial distribution.

Let L and l denote the maximum and minimum localized depth values within the dense group, respectively, yielding the cluster depth variance ΔL=L−l. A critical empirical threshold is set at $\Delta L^{\prime }=24.2\;\mathrm{mm}$, corresponding to the average cap thickness of mature *Agaricus bisporus* measured from 500 randomly sampled mushrooms. The proximity lookup and re-sorting boundary $\left(L^{\prime }=l+\Delta L^{\prime }\right)$ operate based on the following constraints:

If $\Delta L>\Delta L^{\prime }$, significant height differences exist within the cluster, indicating overlapping mushrooms. Mushrooms satisfying $L_{i}\leq L^{\prime }$ are first harvested and sorted in ascending order of depth, thereby prioritizing mushrooms located closer to the camera. The remaining mushrooms are subsequently scheduled using the outside-in radial strategy;

If $\Delta L\leq \Delta L^{\prime }$, the mushrooms are considered to have similar growth heights and negligible vertical overlap. In this case, the dense cluster is scheduled using the same outside-in radial strategy as sparse clusters, prioritizing peripheral mushrooms before proceeding toward the cluster center.

The essential execution procedure is summarized in Algorithm 1.
**Algorithm 1.** Morphology-Aware Hierarchical Harvesting Sequence PlanningInput: Localized mushroom dataset $P=\left\{\left(p_{i},C_{i},L_{i}\right)\left|i=1,2,\ldots ,n\right.\right\}$. Output: Harvesting sequence queue S 1: Construct a 3D KD-tree T using the spatial coordinates of P 2: Partition *P* into three category subsets: $P_{one},P_{sparse},P_{dense}$ 3: Process categories according to the priority: $P_{one}≺P_{sparse}≺P_{dense}$ 4: Sort $P_{one}$ in ascending order of $D_{i}=\|p_{i}-O_{c}{\|}_{2}$ 5: For each sparse cluster: 5.1:  sort mushrooms in descending order of 5.2: $R_{i}=\|p_{i}-P_{c}{\|}_{2}$ (outside-in ordering) 6: For each dense cluster: 6.1:      calculate ΔL=L−l 6.2:      if $\Delta L>\Delta L^{\prime }$ then 6.3:              sort mushrooms with $L_{i}\leq L^{\prime }$ 6.4:              in ascending depth order 6.5:              apply outside-in ordering to 6.6:              the remaining mushrooms 6.7:      else 6.8:              apply outside-in ordering 6.9:      end if 7: Merge all sorted targets into S 8: Return S

By transforming disorganized spatial coordinates into a structured morphology-aware harvesting sequence, the proposed framework facilitates efficient target scheduling while reducing unnecessary manipulator motion and minimizing interference among neighboring mushrooms in dense cultivation environments.

## 4. Control System Design

### 4.1. Overall Design of the Control System

**Figure 13:**
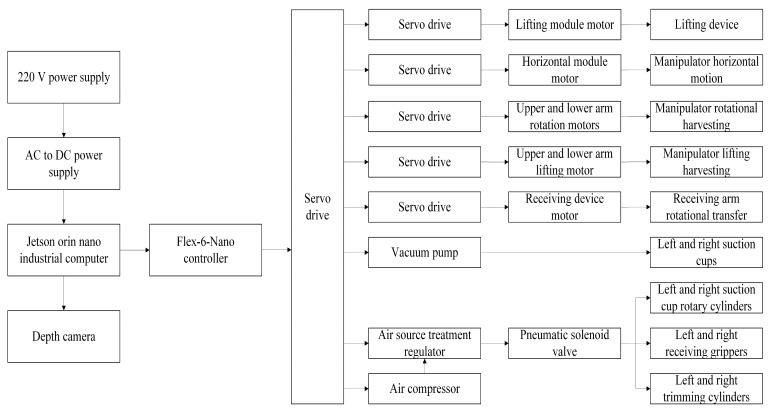
Overall architecture of the mushroom harvesting and grading integrated control system.

The *Agaricus bisporus* harvesting robot is required to perform several key functions. The picking device should be able to automatically move to any mushroom layer. The system should capture complete images of the picking unit while accurately identifying mushroom bodies. It should also complete harvesting, trimming, and grading in an integrated process. Based on these requirements, an integrated control system for harvesting and grading was developed, as shown in Figure 13.

The control process begins when the picking device is activated. A stepper motor driver controls the lifting module motor, moving the device to the designated mushroom layer. Once the system reaches the recognition area, a depth camera captures RGB and depth images of the mushrooms and transmits the data to the industrial computer, Jetson Orin Nano. The industrial computer processes the images using the picking sequence planning algorithm and generates harvesting information, which is then sent to the Flex-6-Nano controller via the Modbus TCP protocol. The Jetson Nano serves as the upper-level controller and is responsible for image processing, mushroom localization, and harvesting sequence planning. After the target coordinates are generated, motion commands are transmitted to the motion controller, which coordinates the operation of multiple motion axes and generates the corresponding control signals for the actuators.

After receiving the commands, the controller uses relay modules to drive each motor, allowing the robotic arm and the horizontal sliding module to move to the target mushroom cap position. At this point, the pneumatic system is activated. The vacuum pump starts, and the suction cup attaches to the mushroom. The lifting motor raises the robotic arm to a specified height, while servo motors drive the upper and lower links to move toward the handover position with the receiving device.

At the same time, the controller drives the servo motor of the receiving mechanism through relay modules, causing the receiving arm to rotate and complete the transfer process. The harvested mushroom is placed onto the receiving gripper. A pneumatic solenoid valve controls the gripper to clamp the stem, and the receiving arm continues rotating to deliver the mushroom to the trimming device. The pneumatic system then actuates the cutting cylinder to remove the stem. Finally, based on predefined grading information, the receiving arm rotates the trimmed mushroom above the corresponding collection box, releases it by reversing the gripper, and completes the entire process of harvesting, trimming, and grading.

### 4.2. Hardware Design of the Control System

The hardware system consists of a motion controller, lifting mechanism, picking mechanism, and receiving and grading mechanism. The lifting mechanism uses a servo motor to control the vertical movement of the sliding table, enabling precise positioning. The picking mechanism includes a horizontal sliding module, robotic arm, arm lifting module, pneumatic control unit, and flexible suction cup, which together execute the picking task according to control commands.

The receiving and grading mechanism is composed of a receiving servo motor, a rotary servo motor, a pneumatic control unit, and two servo drivers. This subsystem is responsible for interaction with the picking arm and subsequent grading operations. The trimming device consists of an air pressure regulating valve, pneumatic solenoid valve, and cylinder, which are used to cut the mushroom stems.

**Figure 14:**
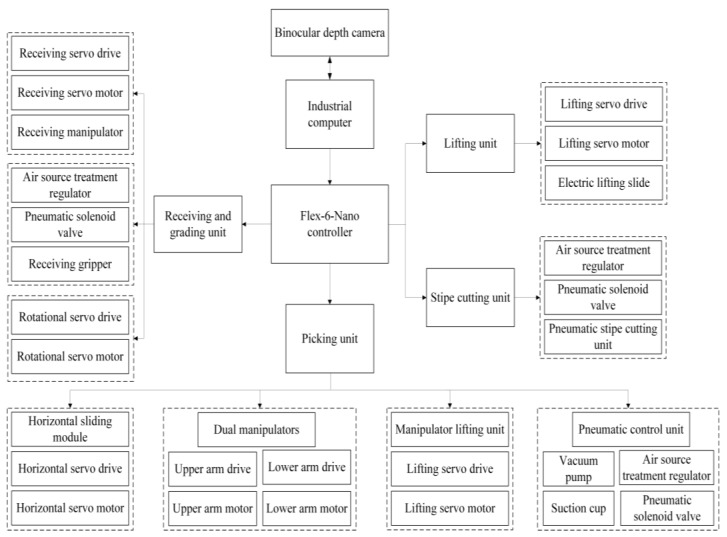
Hardware control system schematic diagram.

These hardware components involve actuators such as servo motors, motor drivers, air preparation units, and solenoid valves. They receive instructions from the industrial computer and execute precise closed-loop control. The overall principle of the hardware control system is illustrated in Figure 14.

#### 4.2.1. Design of the Electric Control System

**Figure 15:**
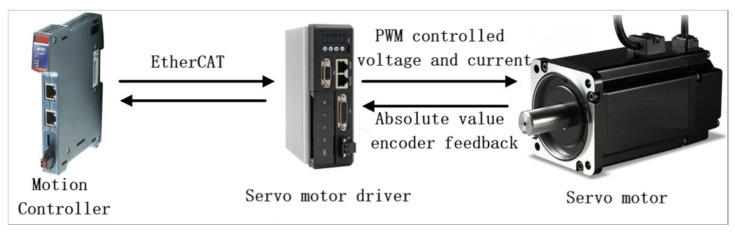
Electric control schematic diagram.

The control system employs a position-based closed-loop control strategy, as shown in Figure 15. The Flex-6-Nano controller sends commands to the servo driver via the EtherCAT protocol, which generates PWM signals to regulate the servo motor’s current and voltage, enabling precise motion control. The EtherCAT protocol features high-speed communication, accurate time synchronization, and flexible network topology, supporting precise synchronized operation of multiple devices and ensuring seamless coordination among components during tasks such as picking, root cutting, and grading. During electrical control, the servo motor communicates with the driver through an encoder to provide feedback on position and speed; the driver then uses closed-loop control to adjust the motor’s movement in real time, ensuring that components like the robotic arm and lateral modules perform these tasks accurately.

The Flex-6-Nano controller, developed by Trio Motion Technology (Tewkesbury, Gloucestershire, UK), is a high-performance EtherCAT controller that supports up to 64 axes and provides strong multi-axis coordination capability along with efficient data processing. Servo drivers and motors serve as the key actuators of the *Agaricus bisporus* harvesting robot. The system adopts the servo driver (MCAC825-M20B-EC, JMC, Shenzhen, China) and the servo motor (60ASM400-5-M17BCH, JMC, China). The driver supports both Modbus and EtherCAT communication protocols, enabling efficient reception of control signals and precise motor motion control.

The Jetson Nano (NVIDIA Corporation, Santa Clara, CA, USA) is responsible for image processing, target localization, and harvesting sequence planning, while motor actuation is performed by dedicated servo drivers and motor controllers. Therefore, the Jetson Nano does not directly supply power to the industrial actuators. Motion commands generated by the Jetson Nano are transmitted to the Flex-6-Nano controller, which coordinates actuator motion through the EtherCAT network. Specifications of the servo drive system are shown in Table 2.

#### 4.2.2. Design of the Pneumatic Control System

The pneumatic actuation system includes four types of components: suction cups, rotary cylinders, receiving grippers, and trimming devices. The pneumatic actuators are arranged symmetrically, with two sets of execution units configured. To improve system integration and reduce pipeline complexity, a dual-channel independent pneumatic control scheme is adopted.

**Figure 16:**
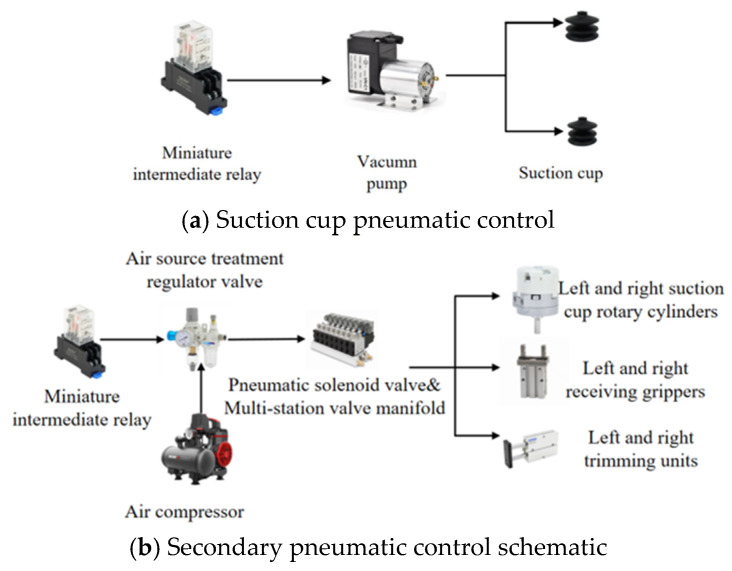
Pneumatic control connection diagram. (**a**) Suction cup pneumatic control. (**b**) Secondary pneumatic control schematic.

The first pneumatic control circuit is dedicated to the negative pressure suction unit of the end effector and is responsible for the adsorption operation during harvesting. This circuit consists of an intermediate relay, two vacuum pumps, air tubes, and left and right suction cups, as shown in Figure 16a. When the control signal is transmitted to the relay, the relay closes and activates the vacuum pumps. Air is extracted through the pipelines, creating a negative pressure at the suction cups, allowing them to attach to the surface of the mushroom.

The second pneumatic control circuit integrates the rotary cylinder, receiving gripper, and trimming device to achieve coordinated control of clamping, posture adjustment, and precise stem cutting. This circuit includes a small intermediate relay, an air source processor with a pressure-regulating valve, an air compressor, an eight-position solenoid valve manifold, and the associated pneumatic components, as shown in Figure 16b. The relay controls the on-off state of electrical signals and connects the air source processor with other electrical components. The air source processor regulates and stabilizes the compressed air while filtering impurities. The air compressor supplies compressed air to the system. The solenoid valve manifold contains multiple independently controlled valves that regulate airflow direction and switching. During operation, compressed air is supplied by the compressor, regulated and filtered by the air source processor, and then directed into the valve manifold. The solenoid valves control the actuation of multiple pneumatic components either independently or simultaneously according to the control signals.

#### 4.2.3. Design of the Visual Localization System

To enable accurate harvesting, the detected mushroom positions in the image must be transformed into three-dimensional coordinates that can be utilized by the robot control system. In the proposed system, the RGB image and depth image acquired by the Gemini 2 depth camera are first fused to reconstruct the three-dimensional point cloud of *Agaricus bisporus*. The spatial position of each mushroom is then obtained in the camera coordinate system using the camera intrinsic parameters and depth information. Subsequently, the reconstructed coordinates are associated with the corresponding predefined recognition region and transmitted to the motion control system for harvesting localization and trajectory execution.

**Figure 17:**
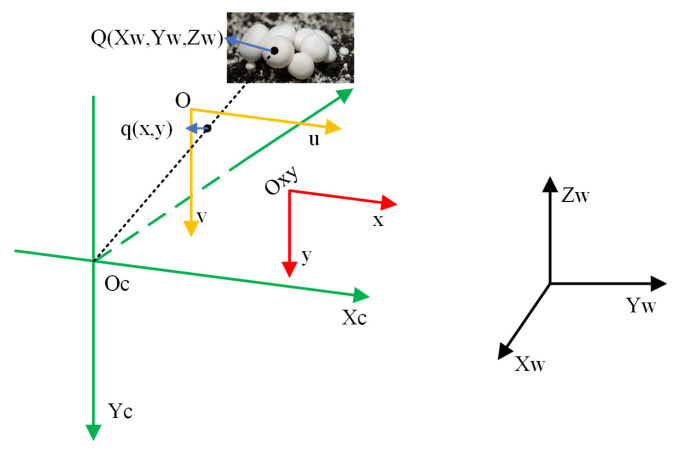
Diagram of the relationships between different coordinates.

Figure 17 illustrates the relationships among the pixel coordinate system, image coordinate system, camera coordinate system, and robot world coordinate system. The pixel coordinate system $\left(O-u-v\right)$ is defined on the image plane with the upper-left corner as the origin, where *u* and v denote the horizontal and vertical pixel coordinates, respectively. The image coordinate system $\left(O_{xy}-x-y\right)$ is established at the intersection of the optical axis and the image plane. The camera coordinate system $\left(O_{C}-X_{c}-Y_{c}\right)$ is centered at the optical center of the depth camera, with the $Z_{c}$-axis aligned with the optical axis. The world coordinate system $\left(X_{w}-Y_{w}-Z_{w}\right)$ is defined based on the robot reference frame and is used to describe spatial relationships within the harvesting robot workspace.

The generation of mushroom point clouds from the segmented mask image and depth image involves two coordinate transformations: pixel coordinates to image coordinates and image coordinates to camera coordinates.

Let the origin $O_{xy}$ of the image coordinate system correspond to the point $\left(u_{0},v_{0}\right)$ in the pixel coordinate system. The physical dimensions represented by one pixel along the horizontal and vertical directions are denoted by $d_{x}$ and $d_{n}$, respectively. The conversion relationship can be expressed as

(1) $$\begin{array}{c} u=\frac{x}{d_{x}}{+u}_{0}\\ v=\frac{y}{d_{y}}{+v}_{0} \end{array}$$

For convenient computation, the relationship can be represented in homogeneous form as

(2) $$\left[\begin{array}{c} u\\ v\\ 1 \end{array}\right]=\left[\begin{array}{ccc} \frac{1}{d_{x}} & 0 & u_{0}\\ 0 & \frac{1}{d_{y}} & v_{0}\\ 0 & 0 & 1 \end{array}\right]\left[\begin{array}{c} x\\ y\\ 1 \end{array}\right]$$

where $\left(u,v\right)$ denotes the pixel coordinates and $\left(x,y\right)$ denotes the image coordinates.

According to the standard pinhole camera model, the three-dimensional coordinates $\left(X_{c},Y_{c},Z_{c}\right)$ in the camera coordinate system can be directly reconstructed from the pixel coordinates (*u*, *v*) and the depth value $Z_{c}$ via the inverse projection transformation, as follows:

(3) $$X_{c}=\frac{\left(u-c_{x}\right)Z_{c}}{f_{x}}\;Y_{c}=\frac{\left(v-c_{y}\right)Z_{c}}{f_{y}}\;Z_{c}=Depth\left(u,v\right)$$

where $c_{x}$ and $c_{y}$ denote the principal point coordinates of the camera, and $Depth\left(u,v\right)$ is the depth value corresponding to pixel $\left(u,v\right)$. The depth values $Z_{c}$ were scaled from millimeters to meters by applying a conversion factor of 11000.

In this study, the intrinsic matrix K and the lens distortion vector D used for three-dimensional reconstruction were extracted directly from the device firmware via the factory calibration of the Orbbec Gemini 2 depth camera, with:

(4) $$K=\left[\begin{array}{ccc} 689.66 & 0 & 644.11\\ 0 & 689.66 & 363.21\\ 0 & 0 & 1 \end{array}\right]\;D=\left[k_{1},k_{2},p_{1},p_{2},k_{3}\right]=\left[0.00125,-0.00214,-0.00015,0.00028,0.00042\right]$$

Specifically, the distortion vector D was applied to rectify the geometric distortion of the RGB images, after which the depth images were spatially aligned with the rectified RGB frame to ensure pixel-level coordinate consistency prior to point-cloud generation. 

After obtaining the three-dimensional coordinates of all pixels belonging to the mushroom mask, a spatial point cloud is generated, and its geometric center $P_{c}={\left[X_{c},Y_{c},Z_{c}\right]}^{T}$ is calculated to represent the target mushroom position in the camera coordinate system.

To transform the target from the camera frame to the robot world coordinate system $O_{w}-X_{w}Y_{w}Z_{w}$ for trajectory execution, a rigid hand-eye extrinsic transformation is applied. The rigid Rotation Matrix R and the Translation Vector T were determined, yielding the following deterministic baseline parameters:

(5) $$R=\left[\begin{array}{ccc} 0.999998 & -0.001396 & -0.002094\\ 0.001396 & 0.999996 & -0.002618\\ 0.002094 & 0.002618 & 0.999994 \end{array}\right]\;T=\left[\begin{array}{c} 0.0025\\ 0.1700\\ -0.2000 \end{array}\right]$$

Consequently, the final harvesting target coordinate $P_{w}={\left[X_{w},Y_{w},Z_{w}\right]}^{T}$ in the robot workspace under the corresponding region is rigorously calculated by applying the partitioned rigid transformation:

(6) $$P_{w}=R\cdot P_{c}+T$$

Based on the obtained $P_{w}$, the motion control system performs inverse kinematics and trajectory planning to complete the harvesting operation.

**Figure 18:**
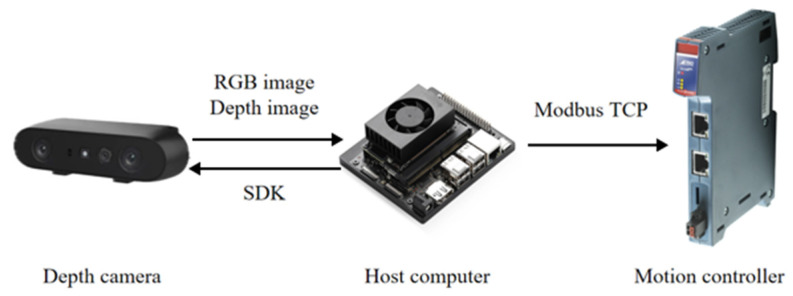
Components of the recognition and positioning hardware system.

The vision-based recognition and localization system of the *Agaricus bisporus* harvesting robot mainly consists of an Orbbec Gemini 2 depth camera, auxiliary lighting, and a Jetson Orin Nano industrial computer, as shown in Figure 18. The depth camera is mounted near the end-effector and equipped with supplemental lighting to ensure stable image acquisition. RGB-D images captured by the camera are transmitted to the industrial computer via a Type-C connection for mushroom detection and three-dimensional position estimation. The obtained three-dimensional coordinates are then associated with the predefined spatial pose of the current recognition region and transmitted to the motion controller through the Modbus TCP protocol, providing positional information for robotic harvesting operations.

### 4.3. Software System Design

#### 4.3.1. Communication Protocol

The integrated harvesting robot adopts a hierarchical communication architecture consisting of a perception layer, a decision-making layer, and an execution layer. The perception layer includes the Orbbec Gemini 2 depth camera and the Jetson Orin Nano industrial computer. The depth camera captures RGB-D images of the cultivation bed and transmits the image data to the industrial computer through a USB Type-C interface. The industrial computer performs mushroom detection, three-dimensional localization, and harvesting sequence planning, generating the target coordinates in the camera coordinate system together with the grading information required for harvesting operations.

Communication between the industrial computer and the Flex-6-Nano motion controller is established through the Modbus TCP protocol. As a standard Ethernet-based industrial communication protocol, Modbus TCP provides reliable data transmission between the upper-level decision-making system and the motion control system. The industrial computer packages the target coordinates and grading information obtained from the vision system into Modbus TCP data frames and transmits them to the motion controller. Upon receiving the data, the motion controller parses the target coordinates and classification information, combines them with the predefined spatial parameters of the current recognition region, and generates the corresponding motion commands through inverse kinematic calculations.

At the execution layer, the Flex-6-Nano motion controller communicates with the servo drives through the EtherCAT protocol. EtherCAT enables high-speed real-time communication and synchronized control of multiple motion axes, ensuring accurate coordination among the horizontal sliding module, lifting module, harvesting manipulator, receiving mechanism, and grading mechanism. The servo drives regulate the current and voltage supplied to the servo motors according to the received commands, while the built-in absolute encoders provide real-time position feedback to form a closed-loop control system. In addition, pneumatic actuators used in the stipe-cutting mechanism are controlled through digital output signals from the motion controller.

Through the coordinated operation of Modbus TCP and EtherCAT communication networks, perception information, target coordinates, harvesting sequence information, and motion commands can be transmitted efficiently throughout the system. This communication architecture enables reliable information exchange among the vision system, motion controller, servo system, and pneumatic actuators, thereby ensuring stable and accurate execution of harvesting, stipe-cutting, and grading tasks.

#### 4.3.2. The Human–Machine Interface

**Figure 19:**
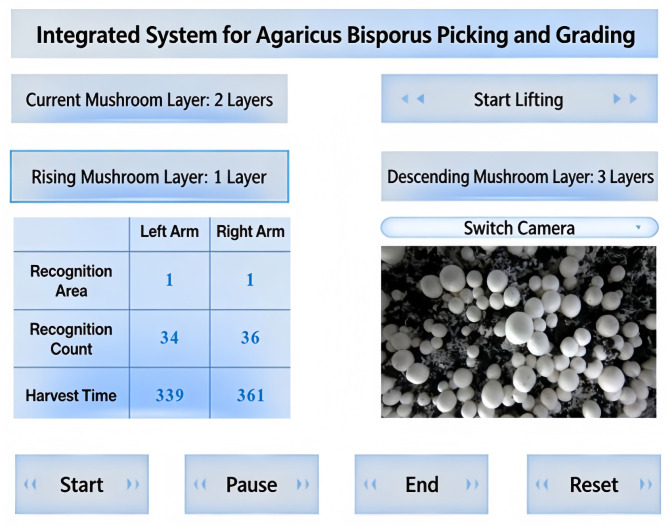
Human–machine interaction interface.

The human–machine interface of the upper computer is developed using QT and is used for operation and monitoring of the integrated harvesting and grading control system, as shown in Figure 19. The interface is divided into three main modules: an image display module, a data display module, and a control button module.

The image display module presents the mushroom images captured by the cameras and allows switching between different camera views. The data display module shows information such as the current detection regions covered by the cameras on both robotic arms, the number of detected mushrooms, and the cumulative system operating time. The control button module includes four buttons: “Start”, “Pause”, “Stop” and “Reset”. When the “Start” button is pressed, the harvesting operation begins. The “Pause” button temporarily halts the current task, and pressing “Start” again resumes operation from the interruption point. The “Stop” button terminates all operations, while the “Reset” button returns all mechanical components to their initial positions.

## 5. System Experiments and Results Analysis

### 5.1. Experimental Environment

**Figure 20:**
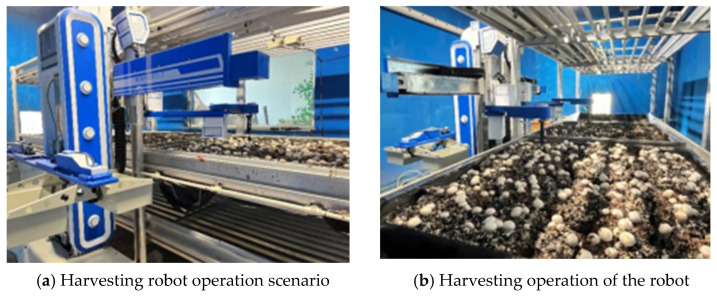
Experimental platform.

The system performance tests were conducted in the *Agaricus bisporus* cultivation laboratory of the College of Agricultural Equipment Engineering, Henan University of Science and Technology. The laboratory was equipped with mushroom cultivation racks measuring 4.5 m in length and 1.4 m in width. Each rack was evenly divided into four cultivation units. During the experiments, lighting, temperature, and humidity were maintained at constant levels to minimize the influence of environmental variations on recognition and picking performance. The harvesting environment is shown in Figure 20.

Before the experiments, system calibration was completed, including extrinsic calibration of the depth camera, zero-position calibration of the robotic arm, and pressure adjustment of the suction and trimming devices. To ensure the reliability of the experimental data, each test was repeated across multiple batches of mushrooms. A total of three groups of experiments were conducted, and the robot harvested mushrooms at four growth stages in each group. Each cultivation unit had a length of 1.4 m and a width equal to that of the cultivation rack.

### 5.2. Evaluation Metrics

For each cultivation unit, the following parameters were recorded: the total number of mushrooms meeting harvesting requirements n, the number of correctly identified mushrooms $n_{1}$, the total number of mushrooms collected in both grading boxes $n_{2}$, the number of mushrooms dropped during interaction $n_{3}$, the number of visibly damaged mushrooms $n_{4}$, the number of misclassified mushrooms $n_{5}$, and the time required for harvesting *t.* Each experiment was repeated three times, and the final performance indicators were obtained by averaging the results.

The recognition accuracy $R_{ar}$ is defined as:

(7) $$R_{ar}=\frac{n_{1}}{n}\times 100\%$$

where *n* is the total number of mushrooms meeting harvesting requirements, and $n_{1}$ is the number of correctly identified mushrooms.

The harvesting success rate $R_{re}$ is defined as:

(8) $$R_{re}=\frac{n_{2}}{n_{1}}\times 100\%$$

where $n_{2}$ is the total number of mushrooms successfully collected.

The interaction failure rate $R_{if}$ is defined as:

(9) $$R_{if}=\frac{n_{3}}{n_{2}+n_{3}}\times 100\%$$

where $n_{3}$ is the number of mushrooms dropped during the interaction process.

The harvesting damage rate $R_{hd}$ is defined as:

(10) $$R_{hd}=\frac{n_{4}}{n_{2}}\times 100\%$$

where $n_{4}$ is the number of visibly damaged mushrooms.

The grading error rate $R_{ce}$ is defined as:

(11) $$R_{ce}=\frac{n_{5}}{n_{2}}\times 100\%$$

where $n_{5}$ is the number of incorrectly classified mushrooms.

### 5.3. Experimental Schemes

- Harvesting Experiment

To evaluate the effectiveness of the picking sequence planning algorithm, multiple cultivation units were selected as test objects. Harvesting experiments were conducted across four growth stages of *Agaricus bisporus*. Differences in density, maturity, and spatial distribution at each stage provided a comprehensive evaluation of the robot’s ability to detect and harvest dense clusters, sparse clusters, and individual mushrooms. During the experiments, the number of detected mushrooms, successfully harvested mushrooms, and damaged mushrooms were recorded to calculate recognition accuracy, harvesting success rate, and damage rate.

- Picking-to-Receiving Transfer Experiment

To assess system stability, picking-to-receiving transfer experiment were conducted. The picking arm placed harvested mushrooms onto the receiving gripper, which then performed trimming and grading operations. At the same time, the picking arm immediately proceeded to the next target, enabling parallel operation. The number of mushrooms dropped during the transfer process was recorded to calculate the interaction failure rate.

- Trimming Experiment

The trimming device adopts a parallel dual-blade structure, and its performance directly affects mushroom quality. To evaluate trimming stability and quality, the success rate of trimming was recorded after mushrooms were transferred to the trimming position. The flatness of the cut surface and the presence of residual stem material were also observed. The removed stems were collected in a dedicated container, while the trimmed mushrooms were directed into corresponding collection boxes based on grading results.

- Grading Experiment

Before the experiment, grading thresholds for categories A and B were defined. During operation, mushrooms were classified based on cap diameter detected by the algorithm, and the receiving mechanism directed them into corresponding collection boxes. After the experiment, the number of mushrooms in each collection box and the number of misclassified samples were recorded to calculate the grading error rate.

### 5.4. Results and Analysis

#### 5.4.1. Harvesting Experiment

According to the above experimental method, three groups of harvesting experiments on *Agaricus bisporus* were conducted, and the results are shown in Table 3, Table 4 and Table 5.

Across the three experimental groups, the robot achieved an average recognition accuracy of 96.64%, harvesting success rate of 95.39%, and harvesting damage rate of 1.63%. These results demonstrate the effectiveness of the vision system, harvesting sequence planning algorithm, and coordinated harvesting mechanism under practical cultivation conditions.

Based on the observed phenomena of recognition failure, harvesting failure, and harvesting damage during the experiments, the causes are analyzed as follows:

- Reasons for recognition failure

Recognition failures mainly resulted from severe occlusion in dense clusters, ambiguous boundaries between overlapping mushrooms, and occasional misidentification of mycelium-covered substrate particles. These factors reduced feature distinguishability and increased the probability of missed or incorrect detections.

- Reasons for harvesting failure

Harvesting failures were primarily caused by inclined mushrooms that prevented effective suction-cup sealing and by reduced suction performance resulting from substrate residue accumulation on the suction cup surface.

- Reasons for harvesting damage

Harvesting damage was mainly caused by two factors. First, in dense regions, the end-effector could collide with adjacent mushrooms that were not detected, resulting in surface damage. Second, during the grading process, mushrooms were released by inversion and subjected to free fall, causing impact with the collection box or other mushrooms and leading to surface damage.

#### 5.4.2. Picking-to-Receiving Transfer Experiment

To evaluate the performance of the transfer process between the picking manipulator and the receiving device, the number of mushrooms dropped during the transfer process was recorded. The results are shown in Table 6. 

The average transfer failure rate was 0.35%,demonstrating the reliability of the transfer process from harvesting to receiving. Failures mainly occurred when inclined mushrooms could not be accurately grasped by the receiving mechanism or when fragile stipes fractured during transfer.

#### 5.4.3. Trimming Experiment

**Figure 21:**
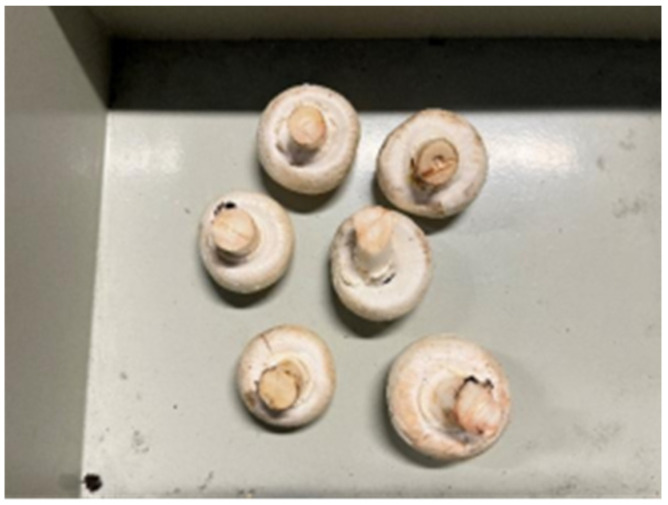
Root cutting effect diagram.

In the integrated harvesting system for *Agaricus bisporus*, the receiving device accurately positions the mushroom at the trimming unit, where root cutting is performed using a pair of parallel dual-blade cutters. This cutter design ensures both precision and efficiency during root removal, and the flatness and smoothness of the cut surface are well maintained, as shown in Figure 21.

As illustrated in Figure 16**.** Root cutting effect diagram, the dual-blade trimming mechanism produced smooth and uniform cut surfaces with minimal residual stipe material. No blockage or additional mechanical damage was observed during the experiments, demonstrating stable trimming performance.

The residual stipe length was controlled by adjusting the transfer height between the harvesting manipulator and the receiving platform before trimming. Throughout the experiments, the trimming mechanism maintained stable cutting performance and produced consistent stipe lengths.

#### 5.4.4. Grading Experiment

The grading of *Agaricus bisporus* is based on the cap circular detection results obtained in the harvesting sequence planning algorithm and classified according to predefined grading standards. To verify the accuracy of the cap circular detection, the diameters of mushroom caps in the two collection boxes were measured, and the number of misclassified samples was recorded.

As shown in Table 7, the grading error rates in the three groups were 1.21%, 1.58%, and 1.04%, respectively, with an average grading error rate of 1.28%. The results indicate that the classification algorithm based on cap circular detection can accurately estimate mushroom size and provide a reliable basis for grading operations.

Analysis shows that grading errors are mainly caused by two factors. Grading errors were mainly caused by diameter underestimation for inclined mushrooms and diameter overestimation resulting from partial occlusion between adjacent mushrooms.

#### 5.4.5. Statistical Analysis of Experimental Replicates

To assess the consistency and reliability of the experimental results, statistical analyses were performed on the three replicate groups. For each performance metric, the mean, standard deviation (SD), and 95% confidence interval (CI) were calculated. The results are summarized in Table 8.

The statistical results in Table 8 show consistently low standard deviations across all evaluation metrics, indicating stable performance over repeated experiments. In particular, the harvesting success rate exhibits the smallest variation (SD = 0.10%), suggesting highly repeatable execution once the target mushroom is correctly detected and localized.

The variations across different performance metrics are consistently low, and each indicator exhibits distinct but controlled sources of fluctuation. Recognition accuracy achieves a mean value of 96.64% with a standard deviation of 0.28%, where the minor variation is mainly associated with fine-grained appearance differences among mushrooms and segmentation ambiguity at instance boundaries. The harvesting success rate reaches 95.39% with the smallest standard deviation of 0.10%, indicating highly consistent execution once the target is localized. In comparison, the harvesting damage rate (1.63% ± 0.12%) reflects the sensitivity of cutting and transfer interactions to slight end-effector alignment deviations. The interaction failure rate remains at a very low level of 0.35% ± 0.04%, demonstrating stable performance in the transfer process between harvesting and receiving stages. Finally, the grading error rate of 1.28% ± 0.27% is primarily influenced by the varying degrees of mushroom body tilt during growth.

Despite these metric-specific variations, all indicators exhibit narrow 95% confidence intervals, further confirming the statistical stability of the system across independent trials. This consistency indicates that the proposed harvesting framework maintains reliable performance even under complex spatial configurations in dense cultivation environments.

## 6. Discussion

### 6.1. Comparison with Existing Harvesting Systems and Approaches

To contextualize the performance of the proposed integrated robot, we compare it with several representative *Agaricus bisporus* harvesting systems reported in the literature. Table 9 summarizes the key characteristics and performance metrics where available. Direct quantitative comparison is inherently limited due to differences in experimental conditions. Nevertheless, the comparison provides a useful benchmark.

As shown in Table 9, the proposed robot achieved a harvesting success rate of 95.39% and a damage rate of 1.63%, demonstrating competitive performance compared with existing mushroom harvesting systems. In addition to harvesting and stipe-cutting, the robot integrates an automated grading function, which is rarely reported in previous studies.

A retrospective analysis of the first-flush harvesting data showed that, compared with a naive nearest-first strategy, the proposed picking sequence planning method reduced the estimated collision rate from approximately 4.2% to 1.63%, highlighting its effectiveness in harvesting dense overlapping mushroom clusters.

Overall, the proposed robot combines high harvesting success, low damage, and integrated harvesting, stipe-cutting, and grading functions within a compact side-mounted architecture, making it suitable for factory cultivation environments.

### 6.2. System Performance and Discussion of Advantages

An integrated harvesting robot for *Agaricus bisporus* was developed, which realized the automatic identification, picking, stipe-cutting and grading within narrow working spaces. The robot adopts a symmetrical structure and operates along the side of the cultivation racks. It mainly consists of a mobile platform, lifting device, picking device, receiving device, trimming device, collection device, and an electrical control system. Results show that the robot achieved an average recognition accuracy of 96.64%, a harvesting success rate of 95.39%, and a harvesting damage rate of 1.63%, along with an average interaction failure rate of 0.35% and a grading error rate of 1.28%. 

Compared with existing mushroom harvesting robots, which are generally limited to single-function or loosely coupled subsystems and lack harvesting sequence planning, the proposed system introduces a more complete integrated workflow. In particular, a harvesting sequence planning strategy is incorporated to optimize target selection order based on spatial distribution, which improves operational efficiency and workflow continuity in dense picking environments.

### 6.3. The Future Work

In addition to the demonstrated performance, several practical limitations remain. The current system has a relatively large overall structure, mainly due to the integration of multiple functional modules required for harvesting, stipe-cutting, grading, and material handling within a constrained cultivation environment. Meanwhile, the harvesting speed still requires further improvement to better meet the demands of large-scale continuous operation, with the primary bottlenecks arising from sequential task execution and motion coordination across different functional units.

Ongoing work is being directed toward addressing these limitations from both mechanical and algorithmic perspectives. From a hardware standpoint, efforts are focused on developing a more compact and lightweight mechanical design by optimizing module layout and reducing redundant structural components. From a system control perspective, future improvements will explore parallelization of certain harvesting operations and optimization of motion scheduling strategies to reduce idle time between consecutive actions. 

## 7. Conclusions

Existing mushroom harvesting robots are generally characterized by single or limited functional modules, low operational continuity, and the absence of explicit harvesting sequence planning. To address these limitations, an integrated harvesting robot for *Agaricus bisporus* was developed, incorporating mushroom identification, harvesting, stipe-cutting, and grading into a unified workflow. The robot adopts a symmetrical structure and operates along the side of cultivation racks, enabling efficient operation within narrow growing environments. In addition, a harvesting sequence planning strategy based on YOLOv8n-USD and KD-tree nearest-neighbor search was integrated to improve harvesting efficiency in dense mushroom clusters.

Experimental results demonstrated that the proposed system achieved an average recognition accuracy of 96.64%, a harvesting success rate of 95.39%, a harvesting damage rate of 1.63%, an interaction failure rate of 0.35%, and a grading error rate of 1.28%. These results indicate that the robot can reliably perform perception, harvesting, transfer, stipe-cutting, and grading tasks while maintaining high operational stability and low damage rates. The proposed system provides a more complete and continuous solution for mechanized mushroom harvesting and offers a practical reference for the development of intelligent harvesting robots in controlled-environment agriculture.

## Figures and Tables

**Table 1:** Structural parameters of the harvesting manipulator.

Parameter	Cultivation Bed Width	Harvesting Coverage Width	Upper Arm Length	Forearm Length	Total Arm Length	Cultivation Layer Spacing	Camera-to-Bed Distance	Arm Material
Value	1400 mm	700 mm	470 mm	350 mm	820 mm	450 mm	350 mm	6061 Aluminum Alloy

**Table 2:** Specifications of the servo drive system.

Component	Model	Communication	Feedback	Function
Motion Controller	Flex-6-Nano	EtherCAT	Real-time feedback	Multi-axis coordination
Servo Driver	MCAC825-M20B-EC	EtherCAT/Modbus	Encoder feedback	Motor control
Servo Motor	60ASM400-5-M17BCH	Encoder interface	Absolute encoder	Joint actuation

**Table 3:** Results of the first group of harvesting tests.

Harvesting Stage	Total Mushrooms/Count	Recognized Mushrooms/Count	Collected Mushrooms/Count	Damaged Mushrooms/Count	Recognition Rate $R_{ar}$/%	Harvest Rate $R_{re}$/%	Damage Rate $R_{hd}$/%
First flush	944	914	870	18	96.82	95.18	2.07
Second flush	792	768	730	12	96.97	95.05	1.64
Third flush	586	568	542	7	96.92	95.42	1.29
Fourth flush	360	350	336	4	97.22	96.0	1.19
Total/Average	2682	2600	2478	41	96.94	95.31	1.65

**Table 4:** Results of the second group of harvesting tests.

Harvesting Stage	Total Mushrooms/Count	Recognized Mushrooms/Count	Collected Mushrooms/Count	Damaged Mushrooms/Count	Recognition Rate $R_{ar}$/%	Harvest Rate $R_{re}$/%	Damage Rate $R_{hd}$/%
First flush	972	936	888	20	96.3	94.87	2.25
Second flush	820	792	754	14	96.58	95.2	1.86
Third flush	602	582	558	7	96.67	95.88	1.25
Fourth flush	416	404	388	4	97.12	96.04	1.03
Total/Average	2810	2714	2588	45	96.58	95.36	1.74

**Table 5:** Results of the third group of harvesting tests.

Harvesting Stage	Total Mushrooms/Count	Recognized Mushrooms/Count	Collected Mushrooms/Count	Damaged Mushrooms/Count	Recognition Rate $R_{ar}$/%	Harvest Rate $R_{re}$/%	Damage Rate $R_{hd}$/%
First flush	896	860	814	16	95.98	94.65	1.97
Second flush	762	734	698	11	96.32	95.10	1.57
Third flush	556	538	514	6	96.76	95.54	1.17
Fourth flush	388	376	362	3	96.91	96.27	0.83
Total/Average	2602	2508	2388	36	96.39	95.22	1.51

**Table 6:** Picking-to-Receiving Transfer test results.

Group Number	Total Number of Mushrooms in Collection Boxes/Count	Number of Dropped Mushrooms/Count	Interaction Failure Rate $R_{if}$/%
1	2478	8	0.32
2	2588	10	0.39
3	2388	8	0.33

**Table 7:** Grading operation test results.

Group Number	Total Number of Mushrooms in Collection Boxes/Count	Number of Grading Errors/Count	Grading Error Rate $R_{ce}/\%$
1	2478	30	1.21
2	2588	41	1.58
3	2388	24	1.04

**Table 8:** Statistical summary of key performance metrics across three experimental replicates.

Metric	Mean (%)	SD (%)	95% CI (%)
Recognition accuracy	96.64	0.28	[95.94, 97.34]
Harvesting success rate	95.39	0.10	[95.14, 95.64]
Harvesting damage rate	1.63	0.12	[1.33, 1.93]
Interaction failure rate	0.35	0.04	[0.25, 0.45]
Grading error rate	1.28	0.27	[0.61, 1.95]

**Table 9:** Performance comparison with existing *Agaricus bisporus* harvesting systems.

System	Harvesting Success Rate	Damage Rate	Intergatred Functions
Shanghai University robot [18]	~85%	~3%	Harvesting + stipe-cutting
Zhong et al. [26]	94.1%	3.57%	Harvesting only
Han et al. [29]	81.8–97.5%	2.1%	Harvesting only
Flexible suction cup only [30]	98.5% (isolated mushroom)	2.5%	Harvesting only
Our proposed system	95.39%	1.63%	Harvesting + stipe-cutting + grading

## Data Availability

The data that support the findings of this study are available from the corresponding author upon request.
